# Association between glycometabolic indicators and risk of early vascular aging and their predictive value

**DOI:** 10.3389/fendo.2026.1946806

**Published:** 2026-09-18

**Authors:** Jinjin Wang, Yujiao Yang, Ruoling Teng, Yi Ding, Fenfen Liu

**Affiliations:** 1 Department of Geriatrics, Mengcheng Second Peoples Hospital, Bozhou, China; 2 Department of Geriatrics, The Third Affiliated Hospital of Soochow University, Changzhou, China

**Keywords:** arterial stiffness, early vascular aging, glycometabolism, insulin resistance, triglyceride−glucose index

## Abstract

**Background:**

Early vascular aging (EVA) elevates cardiovascular risk, and glycometabolic dysfunction is closely associated with arterial stiffness. Seven markers including TyG, CHG and HOMA-series indices were compared to explore their independent correlations and discriminative value for prevalent EVA among physical examinees.

**Methods:**

This retrospective cross-sectional study enrolled 555 participants who underwent baPWV testing, identified from routine health-check records. Baseline data, biochemical indicators and seven glycometabolic indices were collected. Logistic regression, Spearman correlation and ROC analyses were performed to assess associations and diagnostic performance.

**Results:**

Totally 156 participants had prevalent EVA. All seven glycometabolic markers were independently associated with prevalent EVA after confounding adjustment. The CHG index showed strong correlation with mean baPWV (r=0.402, P<0.001). ROC analysis indicated modest discriminative performance for prevalent EVA across all markers; HbA1c yielded the highest observed AUC (0.690), while the TyG index achieved the highest observed discriminative sensitivity (70.51%).

**Conclusions:**

In this retrospective cross-sectional study, all seven glycometabolic indicators were independently associated with prevalent EVA and showed modest discriminative capacity for prevalent EVA within the study sample. The TyG index exhibited the highest observed discriminative sensitivity, while HbA1c showed the highest observed AUC. These two indices may be considered potential low-cost auxiliary markers for identifying prevalent EVA in health-check settings; bootstrap internal validation and prospective external validation are required before broad clinical application.

## 1 Introduction

With the extension of global life expectancy, research on biological aging has attracted increasing attention. Vascular aging interacts with vascular diseases and largely determines individual aging trajectories. Early vascular aging (EVA) describes premature arterial elastic fiber degradation and excessive arterial stiffening, which precedes the onset of hypertension, coronary artery disease and ischemic stroke in young and middle-aged populations (1, 2). Timely screening and intervention of EVA in routine health-check management can effectively reduce the long-term incidence of adverse cardiovascular outcomes.

Brachial-ankle pulse wave velocity (baPWV) is the standard non-invasive tool to quantify arterial stiffness. The 2021 Chinese Expert Consensus on Pulse Wave Velocity Measurement for Early Vascular Aging Evaluation established a unified diagnostic threshold for EVA: age-adjusted baPWV values exceeding 2 standard deviations above the reference mean of healthy individuals (3).

Insulin resistance and combined glucose-lipid dysmetabolism constitute the central pathological cascade accelerating premature vascular aging. Beyond conventional single glycemic markers (FPG, HbA1c), multi-parametric composite metabolic indices including the TyG index, CHG index and HbA1c/HDL-C ratio have been widely validated in recent epidemiological studies (4, 5). These composite indices rely solely on routine blood biochemistry without insulin measurement, making them cost-effective and scalable for primary care and population screening programs. HOMA-IR and HOMA-β are classic steady-state surrogate markers, respectively quantifying systemic insulin resistance and basal pancreatic β-cell secretory capacity, which are fundamental for stratifying glycometabolic dysfunction and cardiovascular risk assessment (6).

Current domestic epidemiological evidence regarding the association between these composite glycometabolic indices and EVA remains scarce, with few head-to-head comparisons of their diagnostic performance for EVA. This retrospective study analyzed cross-sectional physical-examination data from young and middle-aged adults to systematically evaluate the correlation between seven glycometabolic markers and EVA prevalence, and quantify their predictive performance for EVA. Findings of this study will provide empirical evidence for optimized EVA risk stratification and early preventive strategies in population health services.

## 2 Methods

### 2.1 Study population

Participants were adults who completed comprehensive physical examinations including baPWV testing at the Physical Examination Center of Changzhou First People’s Hospital between January 2024 and June 2024. A total of 770 health-check records were initially screened. Participants were excluded if they met any of the following criteria: age <18 years or >60 years (n=75), active infection (n=15), hepatic/renal disease (n=7), malignancy (n=18), hemolytic disease (n=1), autoimmune diseases (n=13), receiving medications interfering with glucose or lipid metabolism (n=25), unclear medication history (n=8), or incomplete laboratory or clinical data (n=53). Duplicate examination records from the same participant were handled by retaining only the most recent complete health-check record. Following these exclusions, a total of 555 residents were ultimately enrolled in the study (**Figure 1**), consisting of 340 males and 215 females.

**Figure 1:**
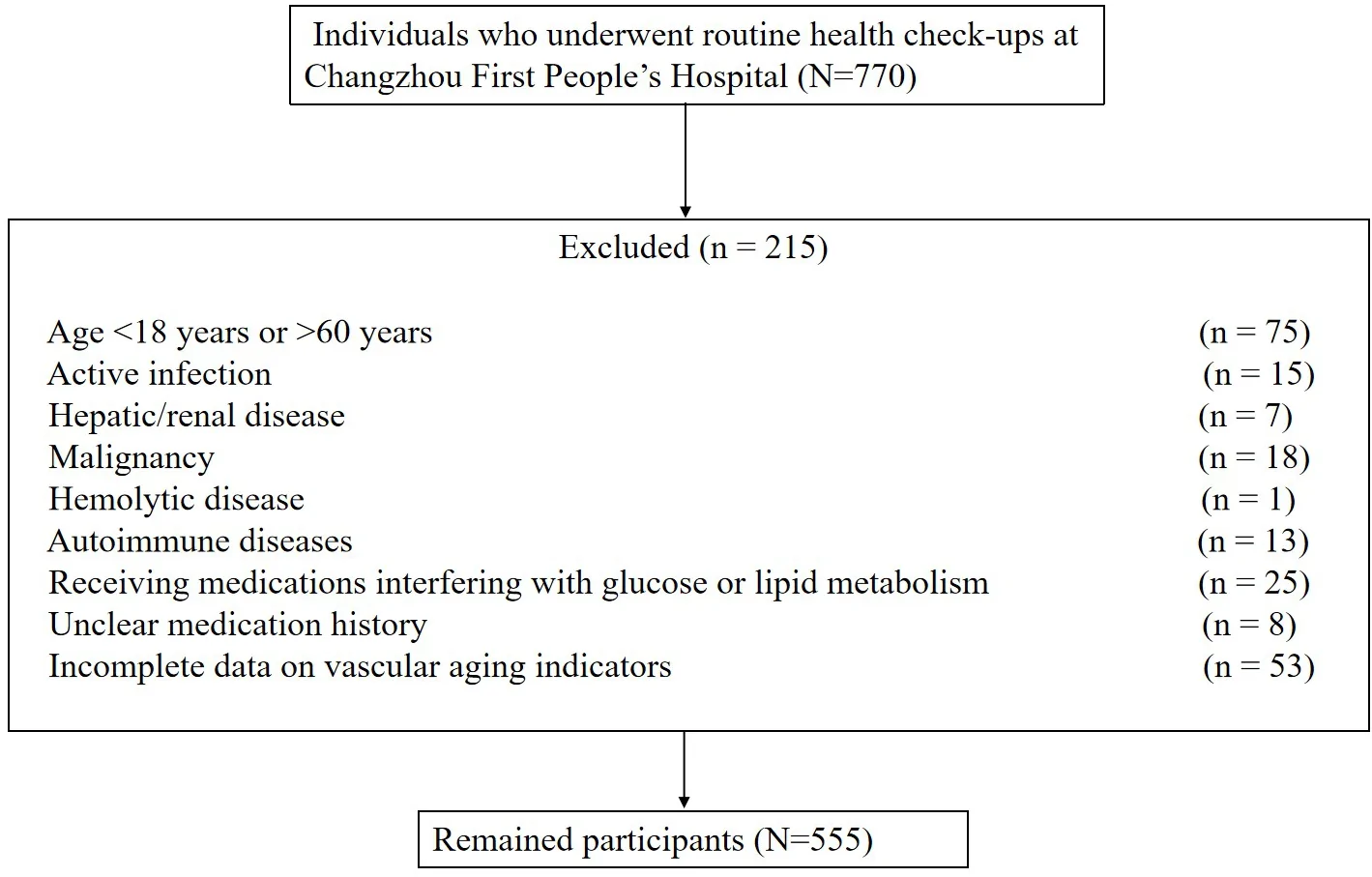
Flowchart of participant selection.

#### 2.1.1 Inclusion criteria

1. Aged 18–60 years old;
2. Completed standardized baPWV examination;
3. Complete clinical and laboratory data available.

#### 2.1.2 Exclusion criteria

1. Age <18 years or >60 years;
2. Severe chronic diseases including advanced hepatic/renal dysfunction, malignant tumors, hematological disorders and autoimmune diseases;
3. Receiving medications interfering with glucose or lipid metabolism;
4. Acute stress status (infection, trauma, perioperative period);
5. Unclear medication history;
6. Incomplete laboratory or clinical data.

### 2.2 Data collection

Baseline demographic data including age, sex, height, weight, body mass index (BMI), self-reported history of hypertension, diabetes, smoking and alcohol consumption were recorded. Laboratory measurements covered FPG, TC, TG, low-density lipoprotein cholesterol (LDL-C), HDL-C, HbA1c, fasting insulin (FINS), serum creatinine, baPWV, and clinic systolic/diastolic blood pressure measured on the day of blood sampling.

### 2.3 baPWV measurement

Brachial-ankle pulse wave velocity was detected using the Omron BP-203RPE III device by certified clinicians following standardized protocols. Participants rested in supine position for 5 minutes before testing. Oscillometric blood pressure cuffs were applied to all four extremities to simultaneously acquire pulse wave signals and blood pressure parameters. Data were automatically processed by integrated software. In accordance with expert consensus (7, 8), EVA was defined as a baPWV value exceeding the age-specific 97.5th percentile of the general healthy population. The baPWV measurements and the age-specific reference percentiles – the latter derived from a database of 7,881 healthy participants – were both obtained using a non-invasive arteriosclerosis detection device (BP-203 RPE III; Omron; Japan) (9).

### 2.4 Calculation of glycometabolic indices

1. TyG index: ln [TG (mg/dL) × FPG (mg/dL)/2].
2. CHG index: ln [(TC (mg/dL) × FPG (mg/dL))/(2 × HDL-C (mg/dL))].
3. HbA1c/HDL-C ratio: HbA1c (%)/HDL-C (mmol/L).
4. HOMA-IR: FINS (mU/L) × FPG (mmol/L)/22.5.
5. HOMA-β: FINS (mU/L) × 20/[FPG (mmol/L) − 3.5]; Participants with FPG ≤ 3.5 mmol/L were excluded for HOMA-β calculation because this value would lead to mathematically invalid denominators.

### 2.5 Statistical analysis

Statistical analyses were performed using SPSS 26.0 and R 4.3.1 software. Non-normally distributed continuous variables were presented as median (interquartile range) [M (Q1, Q3)], and between-group comparisons were conducted via Mann-Whitney U test. Categorical variables were summarized as count (percentage) [n (%)], with intergroup differences assessed by Chi-square test. Multivariable logistic regression models were constructed with EVA status as the binary outcome. The prespecified covariate set included age, sex, measured systolic blood pressure, body mass index, diabetes status, serum creatinine, smoking, and alcohol intake, selected based on established clinical determinants of arterial stiffness. Pairwise Pearson correlation coefficients were assessed before model construction to evaluate multicollinearity; all correlations were below 0.7. Sensitivity analyses were performed by excluding participants with self-reported diabetes, and by excluding participants with both self-reported hypertension and diabetes. We reported odds ratios (OR) and corresponding 95% confidence intervals (CI).

Spearman’s rank correlation analysis was used to quantify linear correlations between each glycometabolic indicator and left, right and mean baPWV, with correlation coefficient r and P-values reported. ROC curves were plotted for each marker to evaluate diagnostic performance for EVA. The maximum Youden index was adopted to determine optimal cut-off values, with corresponding AUC, sensitivity and specificity calculated. DeLong test was performed for pairwise comparisons of AUC values across all indicators. A two-sided P-value <0.05 was defined as the threshold for statistical significance.

For descriptive comparison of effect sizes across glycometabolic indices with heterogeneous numerical scales, we additionally standardized each marker and repeated logistic regression to obtain odds ratios per one-standard-deviation (1-SD) increment. Raw per-unit odds ratios were retained in **Table 1** for reference, while standardized 1-SD OR estimates were reported in the main text only. The inverse association between HOMA-β and EVA risk was explicitly interpreted in subsequent result descriptions.

**Table 1:** Univariate and multivariate binary logistic regression for associations between glycometabolic indicators and EVA.

Indicator	Univariate regression		Multivariate regression (adjusted for confounders)	
	OR (95% CI)	P-value	OR (95% CI)	P-value
HbA1c (%)	2.26 (1.73–2.95)	<0.001	2.29 (1.75–3.00)	<0.001
FPG (mmol/L)	1.59 (1.33–1.90)	<0.001	1.59 (1.33–1.91)	<0.001
TyG index	2.39 (1.79–3.20)	<0.001	2.43 (1.81–3.27)	<0.001
CHG index	3.47 (2.33–5.17)	<0.001	3.12 (2.08–4.67)	<0.001
HbA1c/HDL-C ratio	1.38 (1.22–1.57)	<0.001	1.39 (1.22–1.58)	<0.001
HOMA-IR	1.03 (1.01–1.04)	<0.001	1.03 (1.01–1.05)	<0.001
HOMA-β	1.00 (1.00–1.00)	0.016	1.00 (1.00–1.00)	0.016

EVA, early vascular aging; HbA1c, glycated hemoglobin; FPG, fasting plasma glucose; TyG index, triglyceride-glucose index; CHG index, cholesterol-high-density lipoprotein-glucose index; HOMA-IR, homeostasis model assessment of insulin resistance; HOMA-β, homeostasis model assessment of β-cell function.

## 3 Results

### 3.1 Baseline characteristics of participants

A total of 555 participants were stratified into EVA group (n=156, 28.11%) and non-EVA group (n=399, 71.89%). No significant intergroup differences were observed in age, sex distribution, diabetes history, smoking and alcohol consumption (all P>0.05). The EVA group demonstrated a significantly higher prevalence of hypertension history, elevated systolic and diastolic blood pressure, increased left, right and mean baPWV, higher TC and TG, and lower HDL-C compared with the non-EVA group (all P<0.05). LDL-C levels were comparable between the two groups (P>0.05) (**Table 2**).

**Table 2:** Baseline characteristics of participants stratified by EVA status.

Indicator	Non-EVA group (n=399)	EVA group (n=156)	Z/χ^2^	P-value
Age [M (Q1,Q3), years]	48.00 (40.00,55.00)	49.00 (43.00,55.25)	29007.500	0.213
Sex (male/female) [n (%)]	247/152 (61.90/38.10)	93/63 (59.62/40.38)	0.161	0.689
Hypertension history [n (%)]	75 (18.80)	44 (28.21)	5.348	0.021
Diabetes history [n (%)]	20 (5.01)	12 (7.69)	1.030	0.310
Smoking history [n (%)]	23 (5.76)	14 (8.97)	1.377	0.241
Alcohol consumption history [n (%)]	9 (2.26)	4 (2.56)	0.000	1.000
Systolic blood pressure [M (Q1,Q3), mmHg]	122.00 (112.00,135.00)	134.00 (118.75,146.00)	22241.500	<0.001
Diastolic blood pressure [M (Q1,Q3), mmHg]	74.00 (68.00,82.00)	80.00 (69.75,89.00)	24520.000	<0.001
Left baPWV [M (Q1,Q3), cm/s]	1296.00 (1183.00,1413.50)	1736.50 (1642.75,1902.00)	1069.000	<0.001
Right baPWV [M (Q1,Q3), cm/s]	1300.00 (1195.50,1429.00)	1739.50 (1641.75,1887.50)	1683.500	<0.001
Mean baPWV [M (Q1,Q3), cm/s]	1295.00 (1189.50,1414.50)	1732.00 (1644.25,1908.12)	1167.500	<0.001
TC [M (Q1,Q3), mmol/L]	5.02 (4.48,5.73)	5.24 (4.64,5.88)	27356.500	0.027
TG [M (Q1,Q3), mmol/L]	1.43 (1.04,2.03)	1.90 (1.35,2.52)	22186.000	<0.001
LDL-C [M (Q1,Q3), mmol/L]	2.91 (2.48,3.50)	2.93 (2.54,3.59)	29615.000	0.375
HDL-C [M (Q1,Q3), mmol/L]	1.20 (1.04,1.46)	1.14 (0.98,1.40)	35075.000	0.020

EVA, early vascular aging; baPWV, brachial-ankle pulse wave velocity; TC, total cholesterol; TG, triglyceride; LDL-C, low-density lipoprotein cholesterol; HDL-C, high-density lipoprotein cholesterol.

### 3.2 Intergroup comparison of glycometabolic indicators

HbA1c, FPG, TyG index, CHG index, HbA1c/HDL-C ratio and HOMA-IR were significantly higher in the EVA group, whereas HOMA-β was significantly lower in the EVA group, with all intergroup differences statistically significant (all P<0.05) (**Table 3**).

**Table 3:** Comparison of seven glycometabolic indicators between groups [M (Q1,Q3)].

Indicator	Non-EVA group (n=399)	EVA group (n=156)	Z value	P-value
HbA1c (%)	5.40 (5.10,5.70)	5.70 (5.40,6.20)	-6.511	<0.001
FPG (mmol/L)	4.92 (4.62,5.36)	5.36 (4.92,6.01)	-6.387	<0.001
TyG index	8.63 (8.31,9.04)	9.08 (8.66,9.43)	-6.605	<0.001
CHG index	5.21(5.01,5.43)	5.42(5.20,5.63)	-6.172	<0.001
HbA1c/HDL-C ratio	4.50 (3.64,5.39)	5.09 (4.16,6.12)	-5.126	<0.001
HOMA-IR	13.19 (8.85,19.55)	16.97 (11.55,24.98)	-4.358	<0.001
HOMA-β	805.92 (548.48,1212.58)	747.78 (448.34,1059.71)	2.874	0.004

EVA, early vascular aging; HbA1c, glycated hemoglobin; FPG, fasting plasma glucose; TyG index, triglyceride-glucose index; CHG index, cholesterol-high-density lipoprotein-glucose index; HOMA-IR, homeostasis model assessment of insulin resistance; HOMA-β, homeostasis model assessment of β-cell function.

### 3.3 Logistic regression analysis of glycometabolic indicators and EVA risk

Univariate binary logistic regression confirmed that all seven glycometabolic markers were significantly associated with prevalent EVA (all P<0.05). After adjustment for age, sex, hypertension history, smoking and alcohol intake in multivariate logistic regression, all seven indices remained independently associated with prevalent EVA (all P<0.05). The CHG index also showed a prominent independent association (OR = 3.12, 95% CI: 2.08-4.67, P<0.001) (**Table 1**).

To account for potential residual confounding induced by on-site blood-pressure measurement, adiposity, glycemic status and renal function, we constructed a fuller adjusted model additionally incorporating measured systolic blood pressure, BMI, diabetes status and serum creatinine. After this more comprehensive adjustment, all seven glycometabolic markers still remained independently associated with prevalent EVA (all P<0.05). The TyG index yielded OR = 2.37 (95%CI 1.77–3.32, P<0.001), and the CHG index yielded OR = 4.71 (95%CI 2.70–8.16, P<0.001). Two sensitivity analyses were implemented to verify result robustness. When excluding participants with self-reported diabetes (n=523), the TyG index OR = 2.30 (95%CI 1.70–3.34, P<0.001), the CHG index OR = 4.10 (95%CI 2.35–7.22, P<0.001). When further excluding participants with both self-reported hypertension and diabetes (n=416), the TyG index OR = 2.16 (95%CI 1.55–3.25, P<0.001), the CHG index OR = 3.63 (95%CI 2.06–6.78, P<0.001). The OR magnitudes and statistical significance remained stable across these supplementary analyses, which supported the robustness of the observed cross-sectional associations.

Given that these glycometabolic markers have distinct numerical scales, raw per-unit odds ratios cannot be directly compared for effect-size magnitude. Therefore, we performed additional logistic regression with each marker standardized to per-1-standard-deviation (1-SD) increment for descriptive cross-marker comparison. The standardized 1-SD ORs were: HbA1c OR = 1.81 (95%CI 1.44-2.27, P<0.001); FPG OR = 1.74 (95%CI 1.39-2.17, P<0.001); TyG index OR = 1.70 (95%CI 1.36-2.13, P<0.001); CHG index OR = 1.76 (95%CI 1.41-2.20, P<0.001); HbA1c/HDL-C ratio OR = 1.47 (95%CI 1.18-1.82, P<0.001); HOMA-IR OR = 1.44 (95%CI 1.16-1.79, P<0.001); HOMA-β OR = 0.76 (95%CI 0.62-0.93, P = 0.008). Higher HOMA-β was associated with lower odds of prevalent EVA, indicating an inverse cross-sectional relationship. These standardized effect-size estimates are for descriptive reference only; formal statistical model comparison was not conducted in the present study.

Supplementary univariate analysis was performed for serum uric acid. No significant association was observed between serum uric acid and prevalent EVA (OR = 1.00, 95% CI 0.998–1.002, P = 0.199). Given the non-significant result in univariate analysis, serum uric acid was not further incorporated into multivariable logistic regression, correlation or ROC analyse.

### 3.4 Spearman correlation between glycometabolic indicators and baPWV

Spearman rank correlation analysis showed that HbA1c, FPG, TyG index, CHG index, HbA1c/HDL-C ratio and HOMA-IR were positively correlated with left, right and mean baPWV (all P<0.001). The CHG index exhibited strong correlation with mean baPWV (r=0.402). HOMA-β demonstrated weak negative correlations with left, right and mean baPWV (all P<0.001) (**Table 4**).

**Table 4:** Spearman correlation coefficients between glycometabolic indicators and left, right and mean baPWV.

Indicator	Left baPWV		Right baPWV		Mean baPWV	
	r	P-value	r	P-value	r	P-value
HbA1c (%)	0.372	<0.001	0.381	<0.001	0.377	<0.001
FPG (mmol/L)	0.398	<0.001	0.407	<0.001	0.403	<0.001
TyG index	0.389	<0.001	0.396	<0.001	0.393	<0.001
CHG index	0.391	<0.001	0.414	<0.001	0.402	<0.001
HbA1c/HDL-C ratio	0.293	<0.001	0.302	<0.001	0.298	<0.001
HOMA-IR	0.251	<0.001	0.260	<0.001	0.256	<0.001
HOMA-β	-0.159	<0.001	-0.168	<0.001	-0.164	<0.001

baPWV, brachial-ankle pulse wave velocity; HbA1c, glycated hemoglobin; FPG, fasting plasma glucose; TyG index, triglyceride-glucose index; CHG index, cholesterol-high-density lipoprotein-glucose index; HOMA-IR, homeostasis model assessment of insulin resistance; HOMA-β, homeostasis model assessment of β-cell function.

### 3.5 Diagnostic performance of glycometabolic indicators for EVA

ROC curve analysis was used to evaluate the discriminative ability of each marker for prevalent EVA. HbA1c yielded the highest observed AUC (0.690, 95% CI:0.651–0.728), with an optimal cut-off value of 5.70%, corresponding to sensitivity of 55.13% and specificity of 73.18%. The TyG index achieved the highest observed discriminative sensitivity for prevalent EVA (70.51%) at an optimal cut-off of 8.77, with an AUC of 0.680 (95% CI:0.641–0.719). The ln-transformed CHG index had an AUC of 0.662, optimal cut-off 5.31, sensitivity 64.74%, specificity 62.41%. As an inversely-associated marker, HOMA-β had an AUC of 0.578, optimal cut-off 459.58, sensitivity 26.92%, specificity 84.96% (**Table 5**; **Figure 2**). Pairwise DeLong test comparisons of AUC values showed no statistically significant differences between any pair of indicators except comparisons against HOMA-β (all P>0.05), indicating comparable overall discriminative efficiency across most glycometabolic markers.

**Table 5:** ROC curve parameters for seven glycometabolic indicators predicting EVA.

Indicator	AUC	95% CI	Optimal cut-off	Sensitivity (%)	Specificity (%)
HbA1c (%)	0.690	0.651–0.728	5.70	55.13	73.18
FPG (mmol/L)	0.688	0.649–0.726	5.11	66.67	62.91
TyG index	0.680	0.632–0.725	8.77	70.51	58.40
CHG index	0.662	0.611–0.710	5.31	64.74	62.41
HbA1c/HDL-C ratio	0.630	0.589–0.670	5.25	48.08	71.93
HOMA-IR	0.626	0.586–0.666	20.88	40.38	80.70
HOMA-β	0.578	0.525–0.634	459.58	26.92	84.96

EVA, early vascular aging; AUC, area under the receiver operating characteristic curve; HbA1c, glycated hemoglobin; FPG, fasting plasma glucose; TyG index, triglyceride-glucose index; CHG index, cholesterol-high-density lipoprotein-glucose index; HOMA-IR, homeostasis model assessment of insulin resistance; HOMA-β, homeostasis model assessment of β-cell function.

**Figure 2:**
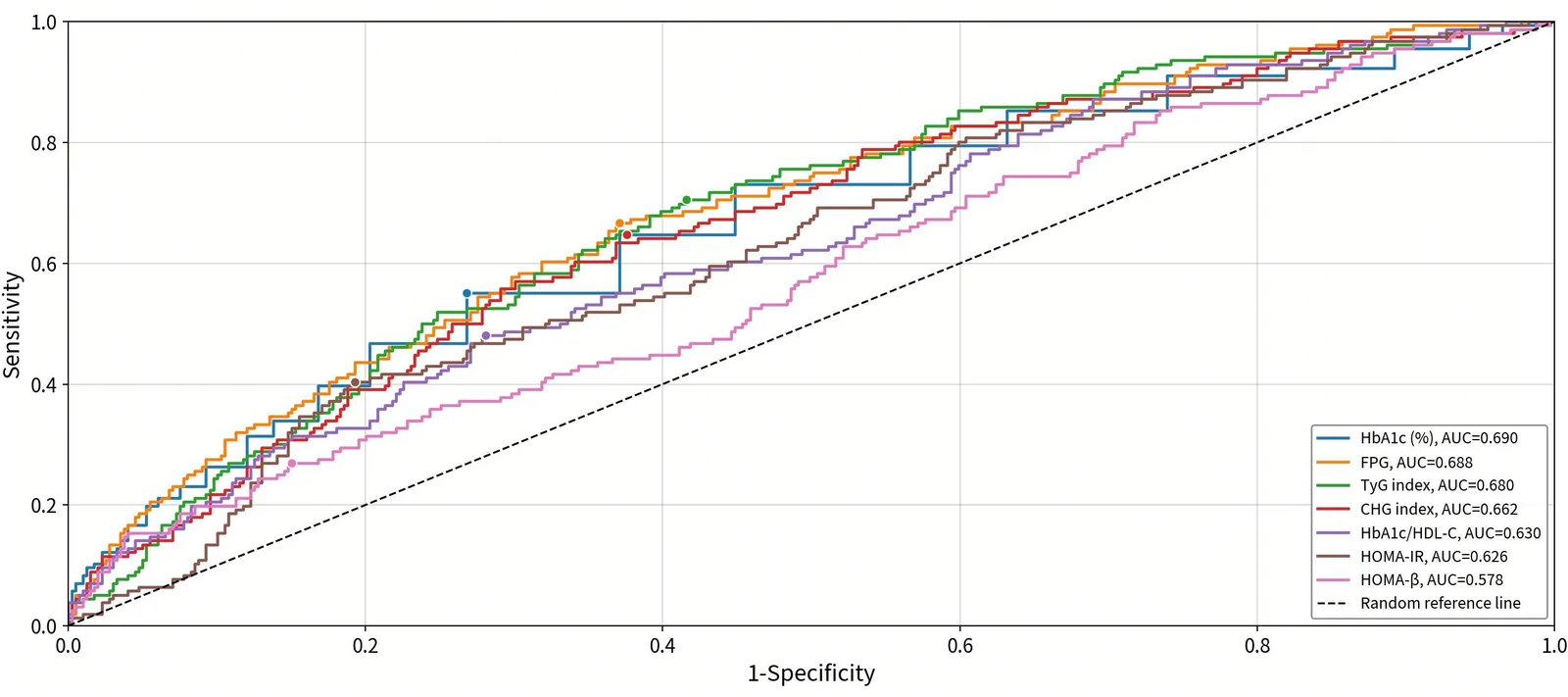
Receiver operating characteristic curves showing discriminative performance of seven glycometabolic indicators for prevalent EVA.

Given that cut-off values derived by maximizing the Youden index within the same analytical sample may produce optimistically biased discriminative estimates, we performed bootstrap internal validation with 1000 resamples to obtain optimism-corrected AUC values and corresponding 95% confidence intervals. After optimism correction, the AUCs were: HbA1c 0.671 (95%CI 0.630–0.711); FPG 0.667 (95%CI 0.627–0.707); TyG index 0.658 (95%CI 0.614–0.701); CHG index 0.640 (95%CI 0.588–0.688); HbA1c/HDL-C ratio 0.611 (95%CI 0.568–0.654); HOMA-IR 0.608 (95%CI 0.564–0.651); HOMA-β (inverse interpretation) 0.592 (95%CI 0.538–0.644). These corrected values confirmed that the glycometabolic markers possess only modest discriminative capacity for prevalent EVA. HOMA-β exhibited inverse discriminative properties: lower HOMA-β values corresponded to higher probability of prevalent EVA. The optimism-corrected estimates are presented for descriptive purposes only; these sample-derived cut-offs still require independent external cohort validation prior to clinical application.

## 4 Discussion

Population aging has driven growing research interest in biological aging mechanisms, with vascular aging forming a bidirectional causal loop with cardiovascular diseases and governing overall individual aging trajectories. This cross-sectional retrospective study systematically analyzed associations between seven glycometabolic markers and EVA prevalence, as well as their predictive performance, based on routine health-check-up data from community adults.

Baseline comparisons demonstrated a significantly higher prevalence of hypertension history in the EVA group (28.21% vs 18.80%, P = 0.021), alongside elevated clinic blood pressure and bilateral baPWV measurements. However, self-reported diabetes prevalence was comparable between groups (7.69% vs 5.01%, P = 0.310). While LDL-C levels did not differ significantly across groups, the EVA group exhibited elevated TC and TG, alongside reduced HDL-C concentrations.

The strong correlation between blood pressure and baPWV aligns with existing clinical evidence (10, 11). Consistent left and right baPWV intergroup differences validated the reproducibility of baPWV testing and confirmed diffuse arterial stiffening among participants with prevalent EVA rather than unilateral localized vascular damage (12). After adjustment for hypertension and other traditional confounders, all glycometabolic indices remained significantly associated with prevalent EVA, suggesting cross-sectional associations between glucose-lipid dysmetabolism and prevalent EVA that are independent of blood pressure (13). This finding highlights that cardiovascular risk assessment in health management should integrate both blood pressure and glycometabolic profiles rather than relying solely on blood pressure readings.

Lipid profile differences yielded important clinical insights. While conventional risk frameworks prioritize LDL-C as the primary atherosclerotic lipid marker, LDL-C levels were comparable between EVA and non-EVA participants in this cohort. Instead, elevated TG and reduced HDL-C were significantly associated with EVA, suggesting that combined hypertriglyceridemia and low HDL-C reflect subclinical arterial stiffening more sensitively than isolated LDL-C elevation in asymptomatic general populations, consistent with recent population-based research (14). Similar diabetes prevalence across groups further demonstrated that subclinical glycometabolic dysfunction (without formal diabetes diagnosis) is sufficient to drive premature vascular aging (15).

FPG and HbA1c were markedly elevated in the EVA group, with positive correlations to bilateral and mean baPWV, acting as independent EVA risk factors consistent with large prospective cohort studies (15). A prospective cohort enrolling over 120 000 healthy adults confirmed that mild elevations in fasting glucose below diabetes diagnostic thresholds independently predict subclinical vascular aging, independent of traditional cardiovascular confounders.

Basic translational research has clarified the molecular mechanisms linking chronic hyperglycemia to vascular aging: sustained high glucose concentrations promote the accumulation of advanced glycation end products (AGEs), which suppress endothelial nitric oxide synthesis and vasodilatory function, stimulate vascular smooth muscle proliferation and migration, and drive arterial wall thickening and remodeling to increase arterial stiffness (16). FPG exhibited a stronger correlation with mean baPWV (r=0.403) compared with HbA1c (r=0.377), implying that fasting glucose reflects acute real-time vascular stress, while HbA1c captures cumulative long-term glycemic burden driving chronic vascular degenerative changes. These two markers may have potential value for auxiliary risk stratification of prevalent EVA in health-check populations, though external validation is still needed before formal screening application (17).

Notably, even participants with normal-high glucose levels demonstrated significant subclinical vascular damage, challenging the clinical paradigm that glycemic risk screening should only target patients with diagnosed diabetes (18). Early lifestyle intervention for individuals with borderline glycometabolic abnormalities represents a critical strategy to delay vascular aging progression. Systematic reviews have comprehensively mapped the molecular cascade linking glycometabolic dysfunction, oxidative stress, inflammation and arterial wall remodeling, further corroborating the pathological pathway observed in this study (19).

Our main findings were maintained after further adjustment for measured systolic blood pressure, BMI, diabetes status and serum creatinine. Sensitivity analyses excluding participants with diabetes or combined hypertension-diabetes also yielded consistent results, demonstrating that the cross-sectional associations between glycometabolic markers and prevalent EVA were not substantially driven by these conventional cardiovascular confounders. Owing to heterogeneous numerical scales across glycometabolic indices, raw per-unit odds ratios are not suitable for direct magnitude comparison. We therefore reported per-1-SD standardized effect sizes for descriptive reference only, without formal model comparison. Among these associations, HOMA-β exhibited an inverse cross-sectional relationship with prevalent EVA, whereby higher HOMA-β corresponded to reduced odds of prevalent EVA. HOMA-IR was significantly higher and HOMA-β significantly lower among participants with prevalent EVA; both markers were cross-sectionally associated with elevated baPWV (20). Pre-existing mechanistic literature suggests combined insulin resistance and progressive pancreatic β-cell secretory decline may be relevant to premature vascular aging. Insulin resistance is regarded as a central mechanistic link connecting metabolic syndrome and vascular aging in pre-clinical research. Experimental data indicate that insulin resistance may activate mTOR signaling in vascular smooth muscle cells to promote proliferation and arterial wall thickening; compensatory hyperinsulinemia may further stimulate the renin-angiotensin-aldosterone system and exacerbate vascular remodeling. Concurrently, impaired endothelial insulin signaling may abolish vasoprotective endothelial function, forming a hypothesized self-reinforcing vicious cycle of hyperglycemia-insulin resistance-vascular injury (21).

Beyond glucose- and lipid-related indices, serum uric acid represents another metabolically relevant biomarker that has been investigated in relation to endothelial dysfunction, oxidative stress and vascular injury. Notably, the clinical relevance of serum uric acid is not restricted to hyperuricemia; both elevated and abnormally low uric acid levels have been associated with adverse vascular and cerebrovascular outcomes. A study of emergency department patients demonstrated a significant association between serum uric acid levels and stroke (22), while another emergency-department-based investigation highlighted the prevalence and clinical significance of hypouricemia (23). In the present cross-sectional health-check cohort, however, univariate analysis showed no statistically significant association between serum uric acid and prevalent EVA (OR = 1.00, 95% CI 0.998–1.002, P = 0.199). This null finding may reflect the relatively healthy study population, the limited range of serum uric acid values, or the cross-sectional design, and does not negate the broader established relevance of serum uric acid in vascular and cerebrovascular disease. Future prospective studies with broader uric acid distributions are warranted to further explore this relationship.

The negative correlation between HOMA-β and baPWV indicates that lower pancreatic β-cell compensation is cross-sectionally correlated with higher arterial stiffness, even among non-diabetic adults. HOMA-β exhibited inverse discriminative properties for prevalent EVA: lower HOMA-β values corresponded to higher probability of prevalent EVA. After accounting for this inverse orientation, bootstrap internal validation yielded an optimism-corrected AUC of 0.592, indicating only limited discriminative information. It cannot be used as an independent discriminative marker for prevalent EVA and may only serve as an auxiliary indicator to grade the severity of underlying glycometabolic dysfunction (24).

The TyG and CHG indices showed modest discriminative ability for prevalent EVA in the present cross-sectional analysis, consistent with contemporary epidemiological research (4, 13). The TyG index requires only routine TG and FPG measurements without insulin testing, rendering it highly implementable in primary care and population health-check settings. In this study, the TyG index achieved the highest observed discriminative sensitivity for prevalent EVA (70.51%) at cutoff value of 8.77. However, bootstrap internal validation yielded an optimism-corrected AUC of 0.658, confirming only modest discriminative performance. Multicenter international cohorts have validated that the TyG index strongly correlates with insulin sensitivity measured via the gold-standard hyperinsulinemic-euglycemic clamp, supporting its utility as a surrogate insulin resistance marker; consistent cross-sectional associations with arterial stiffness have been reported across diverse ethnic populations (13). Whether the TyG index possesses true prospective predictive value for future incident vascular aging requires validation in longitudinal cohort studies.

The CHG index (after natural-logarithm transformation) integrates TC, HDL-C and FPG to simultaneously reflect combined glucose-lipid metabolic dysregulation, capturing cumulative metabolic-related vascular alterations. In this cross-sectional analysis, the ln-transformed CHG index showed strong correlation with mean baPWV (r=0.402). Its ROC cut-off value was 5.31, with sensitivity 64.74% and specificity 62.41%; bootstrap internal validation yielded an optimism-corrected AUC of 0.640, indicating modest discriminative performance. These findings suggest potential for auxiliary cardiovascular risk stratification among high-risk health-check populations (5). A large prospective cohort of over 80 000 Chinese adults reported that elevated CHG index was independently associated with incident cardiovascular events after adjustment for conventional risk factors (14). Whether similar prospective relationships apply to incident vascular aging in our study population remains to be verified.

The HbA1c/HDL-C ratio was also independently associated with EVA, integrating long-term glycemic control and protective lipid status, yet its moderate diagnostic performance limits routine clinical application compared with the TyG and CHG indices (17).

DeLong pairwise AUC comparisons confirmed that all markers except HOMA-β exhibited comparable diagnostic efficiency for EVA, with no statistically significant differences in AUC between HbA1c, FPG, TyG, CHG and HbA1c/HDL-C ratio, consistent with the statistical properties of correlated ROC curves outlined in the original DeLong methodological paper (25).

Current routine health screening protocols primarily rely on isolated blood pressure and single glucose measurements to assess cardiovascular risk, lacking cost-effective composite metabolic biomarkers. Findings from the present cross-sectional study offer preliminary exploratory insights that may inform population cardiovascular risk assessment workflows in health management centers. The TyG index and HbA1c can be integrated into standardized cardiovascular risk evaluation panels for young and middle-aged adults, as both are calculated from routine biochemistry assays without additional invasive testing. For participants with TyG index ≥8.77 or HbA1c ≥5.70, clinicians may consider intensive lifestyle-oriented interventions (weight reduction, glycemic control, regular physical activity) together with serial baPWV monitoring for auxiliary identification of prevalent EVA. Whether such measures can delay progressive vascular aging and mitigate long-term cardiovascular event risks remains a hypothesis requiring prospective validation (16). The CHG index may be considered for auxiliary secondary stratification among screen-positive individuals to identify subgroups that might benefit from more intensive preventive management; this clinical utility also needs prospective confirmation (14).

Crucially, this study identifies elevated EVA risk among asymptomatic adults without diabetes or elevated LDL-C, breaking the traditional screening paradigm focused exclusively on patients with established diabetes or hyper-LDL cholesterolemia, and offering novel frameworks for full-cycle cardiovascular health management in general populations (26). Recent machine learning models incorporating routine glycometabolic markers have also validated their predictive capacity for progressive arterial stiffening, consistent with the findings reported herein (27).

This study carries several inherent limitations. First, it is a single-center cross-sectional retrospective analysis, which can only establish correlational rather than causal relationships between glycometabolic markers and EVA. Large-scale prospective cohort studies are required to validate directional causal effects. Second, the study population was restricted to asymptomatic health examinees, excluding patients receiving glucose/lipid-modifying medications and those with severe chronic illnesses, limiting the generalizability of findings to clinical patient populations with multimorbidity. Future multi-center prospective longitudinal studies with expanded sample sizes are warranted to further characterize the causal relationship between glycometabolic dysfunction and EVA, and develop targeted preventive strategies to reduce EVA prevalence and slow vascular aging progression. Of note, this study is based on retrospective cross-sectional health-check data. Glycometabolic indices and baPWV-defined EVA were assessed at a single time-point. Therefore, we cannot establish temporal sequence or causal relationships between glycometabolic markers and vascular alterations. All reported associations and discriminative performance are restricted to prevalent EVA within this sample. Further large-scale prospective longitudinal cohort studies are required to test whether these markers have true prospective predictive value for future incident vascular aging and cardiovascular endpoints.

## 5 Conclusion

In this retrospective cross-sectional health-check-based study, all seven glycometabolic indicators were independently associated with prevalent EVA and exhibited modest discriminative capacity for prevalent EVA among the study population. The TyG index showed the highest observed discriminative sensitivity for prevalent EVA, while HbA1c yielded the highest observed AUC within this sample. These two readily-accessible composite glycometabolic markers may serve as potential low-cost auxiliary markers to identify prevalent EVA in routine adult health-check-ups for cardiovascular risk stratification in population health services. Prospective longitudinal studies are needed to verify whether these markers can predict future incident vascular aging and related cerebro-cardiovascular outcomes, and to confirm the clinical benefit of marker-guided targeted intervention.

## Data Availability

The raw data supporting the conclusions of this article will be made available by the authors, without undue reservation.
